# Treatment of Septic and Putrescent Teeth

**Published:** 1907-07

**Authors:** C. B. Colson

**Affiliations:** Charlestown, S. C.


### TREATMENT OF SEPTIC AND PUTRESCENT TEETH.

By Dr. C. B. Colson, Charlestown, S. C.

I do not wish you to look upon what I here read as a finished essay, it is only a message, an idea, a fact of something good that it has been my great fortune to fall in with, and I am here my brothers to pass it on. I will endeavor to make the message short and too the point, but it is not the less valuable. So valuable that I could exclaim "Eureka!" "I have found it," but that would be partly false, for I did not find it, it was passed on to me, I have endeavored, with others, to perfect it.

If there is any one present that has a rational or empirical treatment or potent drug that he can, at one sitting, treat and restore to a good aseptic condition a putrescent tooth, or at two sittings, let him hold up his hand I want to see him. Well, I am going to hold up my hand, for I have such, and it works successfully for everybody.

The treatment of septic root canals with their several complications has been the saddest part of our dental history. I will not go into it. There is but one reason, the absolute impotency and non-adaptability of our germicidal agent, for the treatment is nothing but a germicidal treatment.

I will not here mention the various methods or drugs that give us various successes, but only state that we vary greatly and individually get quite different results from the same agents and methods.

I am going to tell you of a simple method and a simple preparation that every man who simply will follow the directions will get the same results and those results will be pleasing and most satisfactory.

Formaldehyde is the germicide to which I want to call your attention and a special preparation of it, non-proprietary, and a happy method of using it.

Many of you have used it in various forms, many of you are using it now, in certain secret and proprietary forms sold and pushed on the market. I do not approve of these proprietary and secret preparations that make you when you use them an empiric and a quack, you know not what you are. The rascals that make them deceive you, they know that it is formaldehyde, that is the potent drug that is the power of their preparation, but they are so modest. (knavishly so) as to barely mention the fact of its presence while giving its formula to pacify your inquiry as to what it is.

Formaldehyde for the past ten years has been tried and experimented with in dentistry and never became popular. Five one-hundreds of its power and its happy adaptability we were ignorant of. Through this the experimenters had many unpleasant experiences with their patients, causing them great pain and the results were invariably poor. I believe the honor of discovering this happy method belongs to Dr. Buckley, of Chicago. He described it in a paper read at the International meeting at St. Louis in 1904. Dr. Buckley found that by putting equal proportions of incresol and formalin together and sealing them in the chamber of a putrescent or septic tooth that the results were invariably happy and satisfactory.

Incresol is the name of three forms of carbolic acid combined. Formalin is the commercial form of formaldehyde U. S. D. and is known as the 40 per cent. standard preparation.

Experience proved to us that the preparation of incresol and formalin although happily successful was not stable, it separated, it did not keep mixed. Other investigators entered the field and what I wish to give to you today is the best we have to date. We have a fairly stable formula that holds together and gives us such results that I can not conceive any improvement and makes me so enthusiastic that, knowing the suffering brothers who need it, I am most eager to give it to you and impress on you its value.

All septic and putrescent teeth, that is the pulp chamber and not canals, being foul, it matters not if the tooth has fistula or a blind abscess, inflamed or not, dry inviduous or putrescent and odorous enough to give the entire office some of its terriffic odors. The simple sealing of the following formula on a saturated piece of cotton within the chamber of the tooth gives all the happy results that none have had before. (Mind you I said chamber not canals.)

The formula is this: Take 2 drachms each of formalin and creosote, this combination forms a slightly cloudy mixture. Shake well and add pure alcohol slowly until it clears. This will take about a drachm of alcohol. If the mixture is clear at first add the alcohol anyhow.

This will give you a stable preparation that will remain so if kept from the light and tightly corked. The preparation should be renewed about once a month as it is a well known fact that formaldehyde is a gas and will escape if allowed to do so.

In opening up such teeth in preparing for the treatment use as little instrumentation as possible, just open the chamber and remove all or a portion of its contents using care not to shove any of the septic contents through the apical foramen.

This preparation should always be sealed. This is very important. I said sealed and sealed with soft cement and nothing else, so as to give no pressure. You do not wish to drive the liquid preparation into the canals at any time. This liquid preparation gives off formaldehyde gas. This reaches everywhere, it will pass up the canal although it may be full of pulp remains, it will enter the tubuli and destroy all germs and spores, it will go beyond the apical foramen and make inactive the contents of the little sack that has been caused by the mphitic gas pressure. It will reach any point of the roots where there are germs forming from this septic canal. Its effect is painless, one treatment seems to be all that is needed and time for nature to restore the parts and close the fistula, but two or more treatments to make doubly sure can be followed. The first treatment should be left in about two days unless there is considerable pus flowing through the canals and in this case I renew the treatment the next day to get rid of the last of the pus.

There is one caution needed here, it seems to make the pus so infectuous that a small quantity may remain outside the apical foramen and the tooth lose all of its soreness and seem perfectly well, but on the passing of a broach to the apex of the foramen it will deliver this pus, that if you had not taken this precaution you would have filled and left in the tissues.

Again a caution. Formaldehyde is never indicated about live pulps, half devitalized pulps or on the stumps of freshly extracted pulps. Only in purely septic and putrescent conditions.

The sealing should be done only with soft cement. A box of cheap Weston of a special color should be kept for these treatments so you can readily know the treated cavity. Never attempt it with stopping or gutta percha as you cannot insert without some pressure. You will waste your time to use sandarac or other gum preparations on cotton as they allow the gas to come out instead of going up and searching and the pressure on same in biting often drives the liquid up the canals and causes pain.

